# Evaluation of Non-Pharmacological Measures Implemented in the Management of the COVID-19 Pandemic in Romania

**DOI:** 10.3390/healthcare10091756

**Published:** 2022-09-13

**Authors:** Nenssy Georgiana Horga, Daniela Cirnatu, Nilima Rajpal Kundnani, Elena Ciurariu, Simona Parvu, Andrei Lucas Ignea, Claudia Borza, Abhinav Sharma, Stelian Morariu

**Affiliations:** 1 Faculty of Medicine, ‘Victor Babes’ University of Medicine and Pharmacy, 300041 Timisoara, Romania; 2 Faculty of Medicine, ‘Vasile Goldis’ University of Medicine and Pharmacy, 310025 Arad, Romania; 3 Department of Functional Sciences, Physiology, Centre of Immuno-Physiology and Biotechnologies (CIFBIOTEH), University of Medicine and Pharmacy ‘Victor Babes’, 2 Eftimie Murgu Square, 300041 Timisoara, Romania; 4 Civil Medical Society Dr Rosca, 307405 Teremia Mare, Romania; 5 Faculty of Medicine, ‘Carol Davila’ University of Medicine and Pharmacy, 050474 Bucharest, Romania; 6 Department of Functional Science, Discipline of Pathophysiology, “Victor Babes” University of Medicine and Pharmacy, 300041 Timisoara, Romania; 7 Center for Translational Research and Systems Medicine, “Victor Babes” University of Medicine and Pharmacy, 300041 Timisoara, Romania; 8 Centre of Cognitive Research in Pathological Neuro-Psychiatry NEUROPSY-COG, “Victor Babes” University of Medicine and Pharmacy, 300041 Timisoara, Romania; 9 Department of Cardiology, “Victor Babes” University of Medicine and Pharmacy, 2 Eftimie Murgu Square, 300041 Timisoara, Romania; 10 Department of Occupational Health, Faculty of General Medicine, “Vasile Goldis” Western University of Arad, 310025 Arad, Romania

**Keywords:** face masks, handwashing, COVID-19, pandemic

## Abstract

The management of the COVID-19 pandemic in Romania has included the involvement of not only the medical system, but also that of the administrative and social services. All these organizations are working together to lower the impact on the health of the general population, to increase the health system’s response capabilities and even to diminish the negative effects upon the economy due to the epidemic’s length. Therefore, non-pharmacological measures (NPMs) imposed through restrictive measures (administrative, economic and individual) have influenced the evolution of morbidity and mortality. Even from the first months of the pandemic’s progression, researchers have shown the impact of the NPMs’ existence, as there were many studies on all NPMs in conjunction, as well as those targeting specific measures such as school closures. Our study started by establishing a temporal relationship between the non-pharmacological measures found in most countries (wearing a mask, washing hands and physical distancing, limiting economic activities, closing schools, limiting internal and international movement, banning public and private events in closed spaces) and the evolution of the pandemic in Romania. The degree of novelty brought by this study consists of extending the analysis to the pre-existing state of the health system and to the measures meant to increase the resilience of the population, as well as to the measures aimed at reducing the type of risk, and factors that can equally influence the evolution of the number of cases. The results of the statistical analysis show the important effects of certain NPMs (mask mandates, online schooling, decisions regarding imposing or lifting local restrictions) as well as the reduced impact of other measures (hand disinfection, social distancing or the restriction of public and private events). Hence, it can be concluded that during such pandemics, implementing quick, simple measures can prevent the spread of the disease and help fight the contagion in a better manner.

## 1. Introduction

The COVID-19 pandemic caused by SARS-CoV-2 left the entire world devastated. Since the first case detected in 2019, it took very little time for this virus to infect a huge number of the population worldwide. Various pharmacological and non-pharmacological measures were implemented by countries around the world [1,2,3]. World Health Organization advisories were issued to limit the spread of the contagion. Various studies have advocated for non-pharmacological measures to reduce the rate of reproduction of COVID-19 cases [4,5,6,7,8,9,10,11]. Mathematical calculations were performed to identify the effects of imposing and lifting measures on the number of COVID-19 positive cases, whereas other methods to predict the evolution of the epidemic crisis were considered, such as the exposome [12] or Index c [13]. However, none of the measures were found to be 100% effective. Inventing a vaccine for a virus with such a short notice was challenging, and to test it on humans was a bigger challenge. Worldwide, the healthcare systems were not prepared for such an outbreak. Various non-emergency operative procedures and routine hospital admissions were put on hold in order to meet the demand of the high number of COVID-19 cases, leaving no spare medical and surgical staff. Surgical guidelines were changed in order to minimize the risk of contracting the virus during the surgery [14]. Special attention was required for critically ill patients who were more prone to exhibit added complications. An increase in microvascular permeability as a systemic inflammatory response due to surgical trauma in combination with a COVID-19-stimulated inflammatory response can be fatal [15]. Testing to detect COVID-19 was implemented on a large scale. Symptoms varied from patient to patient, ranging from mild respiratory disease to the extent of multiorgan failure resulting in death [16,17]. The quality of life (QOL) of people was highly affected, especially in patients suffering from chronic illnesses such as atrial fibrillation, heart failure, multiple sclerosis, oncologic patients, etc., not just due to the difficulty of performing routine investigations, but also due to lockdown-induced anxiety and depression [18]. The advisories and continuous news regarding the rising death toll due to the deadly virus also affected the QOL of the healthy population, leaving a vast number of people with life-long psychological trauma and distorted families. Some treatment protocols required proper dose adjustments, especially in patients suffering from diseases where anticoagulant therapies were already in treatment regimens or in cases where the patient had high bleeding tendencies [19,20,21].

The aim of this study is to evaluate the non-pharmacological measures implemented in the management of the COVID-19 pandemic in Romania, and to understand to what extent the infrastructure of the health system and health regulatory actions with applications at the level of all health units and for the entire population determines a modification in communal and individual behavior, such as at the level of health organizations, in order to be able to control, epidemiologically, the situation in the case of an infectious disease.

Within this study, the period preceding the vaccination of the population is analyzed (March 2020–December 2020) with regard to the ways restrictive, non-pharmacological measures in the health sector have influenced the evolution of the SARS-CoV-2 epidemic in Romania.

Understanding the current working structure of the Romanian healthcare system and measures taken to combat the COVID-19 pandemic by Romania: 

The Ministry of Health represents the central authority within the field of public healthcare and has subsidiary control over the public health departments, the “Institutul National de Sanatate Publica” (The National Institute of Public Health), and the county hospitals and health units with beds, which are considered a national interest. Legea nr. 95/2006 (Law nr. 95/2006) created the framework for the proper sustainable dimension of the pyramid in regard to the health services, establishing as a base for the system community healthcare services, such as those provided by family doctors and ambulatory care, with hospital services following behind having a smaller impact. In addition, the law of this framework, together with the continued decentralization process from 2010, made relations between regulators, financiers and suppliers more complex. In this way, MS and CNAS, the main actors within the supply system of public services within the health domain, play as much a role in supply as the authorities which regulate, finance, accredit and control the public service. This style of fragmented regulation and administrative organization leads to the dispersion of attributes in various normative actions, with the complementarianism of the attributes being difficult to follow, as outside the primary legislation there exists also a secondary legislation composed of an impressive number of orders from the Ministry of Health. Medical services are supplied in Romania based on three pillars of healthcare: primary, ambulatory and hospital. Primary healthcare is assured through individual medical clinics (family doctors) and permanent centers. Ambulatory healthcare is assured through public or private units: individual medical clinics, hospital ambulatory services and polyclinics.

**Figure 1:**
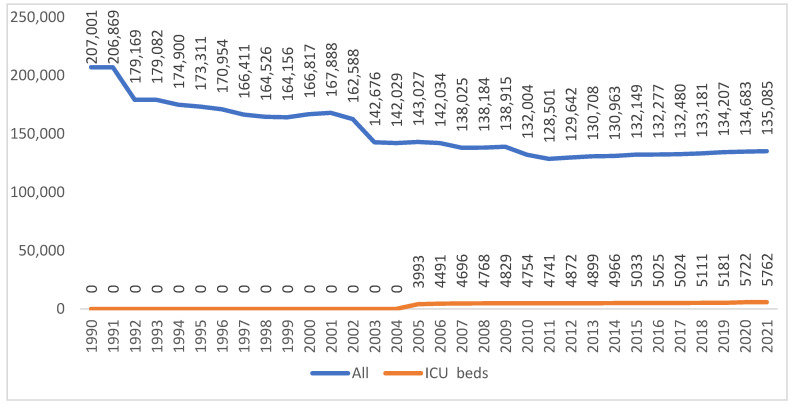
The evolution of the number of beds within hospitals and those from intensive care units in Romania within the period of 1990–2021. (http://statistici.insse.ro:8077/tempo-online/#/pages/tables/insse-table accessed on 20 July 2022).

Of interest for the studied topic is the evolution of the number of hospital beds, and in particular, the number of beds designated for intensive care units (Figure 1). We can observe that although the number of total beds has been reduced by 34.94%, the number of beds for intensive care has risen by 30.22%, an evolution which has allowed for a better response in health units to the necessary healthcare required for the forms of SARS-CoV-2. The acquisition of beds for intensive care units was assured through human resources as related to the required supplement; this was one of the biggest challenges with which all medical systems were confronted with, even from the first weeks of the pandemic’s course, and also for each epidemic wave.

For the management of the epidemiologic situation, on 29 January 2020 the National Committee for the State of Emergency, “Comitetul Național pentru Situații Speciale de Urgență (CNSSU)”, was activated, which is a part of a national system for the management of emergency situations and a technical-scientific support group for the management of highly contagious diseases within the territory of Romania—a technical support body. Documents adopted by the technical-scientific support group are forwarded for approval to the CNSSU, a body able to make decisions in such situations. To facilitate access to information, a web portal was created to display the normative actions and to post official information.

The first measures were taken on 1 February 2020, after the declaration of a pandemic, and aimed to quarantine and accommodate people who entered Romania from affected areas for a period of 14 days at specific locations according to local authorities, with the cost of such accommodations and other related services (personal hygiene products, meals, waste disposal, cleanliness of the residence, etc.) being supported by the state budget. For individuals in quarantine and those who would be isolated in their own residences, the “Institutul National de Sanatate Publica” (National Institute of Public Health) prepared informative material with recommendations [22]. For medical personnel, methodologies and guidelines were provided [23]. Additionally, from the INSP (NIPH) a free telephone helpline was established for supplementary information in regard to the new form of infectious coronavirus disease. The first patient who tested positive for COVID-19 was on 4 March 2020. Measures taken at that moment included the confinement of a positive individual until they had two negative test results, the isolation of individuals who were in contact with an infection case through epidemiological investigation, and PCR tests. The management of COVID-19 PCR positive results in personnel, as well as in patients, was adapted throughout the length of the pandemic according to the scientific information available and published protocols at the international level. Along the evolution of the pandemic, the hospitalization of patients went from all positive patients being hospitalized, to the hospitalization of only symptomatic patients, to finally, only patients presenting pulmonary lesions or other risk factors towards their wellbeing. The testing strategy was periodically adapted, progressing from testing all people in contact with an infected individual to testing only specific categories of people considered at risk. In terms of administrative measures which increased response capabilities, within the first months of the pandemic, medical personnel who provided care to COVID-19 positive patients were given accommodation in order to reduce the possibility of contamination from and within their familial sphere. Likewise, for medical personnel and social service members from medico-social and social service institutes, bimonthly testing of staff was established as procedure. Working hours were similarly modified for this category of employees, with 14-day shifts in which connections with the environment outside of the workplace were greatly diminished; in medico-social and social service institutes, visitors and non-staff individuals were banned from entering.

Through the course of the pandemic the pressure on the healthcare system rose enormously. There was pressure not only on primary healthcare, but also on specialized healthcare, which affected the non-COVID-19 pathologies. Both human and material resources within the healthcare system were directed with preponderance in order to properly provide the care required for patients positive with COVID-19. In medical units with beds, electrical circuits were redesigned, often multiple times, in order to adapt them to the pathogen. Field hospitals or mobile intensive care units were also set up. Given the growing tendency of the number of intensive care-interned COVID-19 patients, for each wave of the pandemic there was a necessity for supplementary measures in order to increase the number of beds for this segment. The significant increase in the number of intensive care beds from the years before the pandemic facilitated the modification to fulfill the requirements of the medical services.

From a legislative point of view for the management of the “COVID-19 situation”, various government decisions, military ordinances and orders from the commander-in-chief were issued. These acts have regulated the measures which increase response capacity, the measures which increase community resistance and even those which decrease the impact of the type of risk on different segments of public life (school, cultural activities, religious gatherings) and economic sectors affected by the pandemic or recreational activities [24]. The non-pharmacological measures which were imposed were derived from the recommendations of the WHO [25] and the EU CDC, yet they were adapted according to the response capabilities of the Romanian healthcare system. In order to increase the response capabilities of the healthcare system, on 23 March 2020 surgical interventions and other non-emergency treatments and investigations were suspended. The rate of infectiousness, which was greater than other pathogenic agents, coupled with airborne transmission leads to a rapid infection in exposed populations in the absence of control measures, creating the possibility of generating extensive outbreaks within communities and overloading or exceeding the abilities of the healthcare system.

In terms of the reactions of civilian society, protests against individual or collective protection measures were recorded in multiple European countries, yet measures instituted through normative acts were maintained. In Romania during the pandemic, both the normative acts issued to manage the spread of COVID-19 as well as previous normative acts which determined the general methods for measures used to combat infectious diseases were contested in the Constitutional Court (Curtea Constitutionala). The contestation that occurred proposed that certain provisions of the law concerning certain measures for preventing and combating the effects of the COVID-19 pandemic were unconstitutional. During the periods where certain measures for limiting the effects of the pandemic, imposed by the government and the Health Ministry through normative acts, were put on pause, the number of cases identified had risen over the original estimated figure. In this way, within the period of 3/06–15/06, a significant increase in the number of new cases and of severe cases in IC wards, and in the average rate of positive cases calculated as a rolling average, with values from 1.7% to values of 2.8%, were observed. Additionally, in this period, there was once again a new sub-unitary report that recorded the number of recovered patients and the number of confirmed patients after approximatively a month of increased numbers. This trend towards growth could be explained through the dynamics between the relaxation of sectorial measures and through the low degree of compliance in respecting social distancing measures.

Beginning on 15 September 2020, an acceleration in the growth rate was recorded; this became much more prominent in the period between 5 October and 19 November, followed by a period where the rate decreased until 30 December 2020. The drops in cases that are prominent around the National Day of Romania and the winter festivities were generated by a decreased tendency for individuals to test themselves when they had minor symptoms (preferring to spend these mini-holidays at home and to test themselves after they had ended). These drops were adjusted during the following periods, with an observed increase of 7–10 days of growth after each mini-holiday.

Community resilience varies from one community to another, yet the growing pressure on healthcare services has forced health facilities to adapt their ability to care to the detriment of other categories of patients, with the number of supplementary deaths being directly correlated to the increase in the number of cases. Contradictory information, the spread of false news, and inadequate protection measures or even neglect were factors which have shaped the response of individuals. These occurrences have been encountered in most countries [26,27]. 

## 2. Materials and Methods

**Figure 2:**
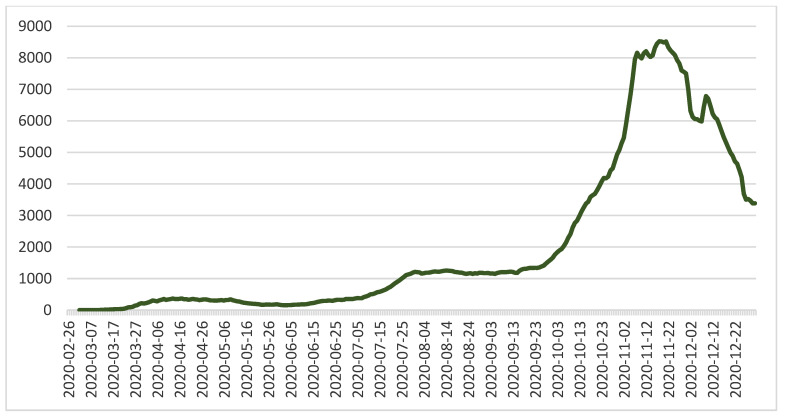
The evolution in the number of individuals tested positive (new cases smoothed) with COVID-19 in the period of March 2020–December 2020 in Romania [28].

**Figure 3:**
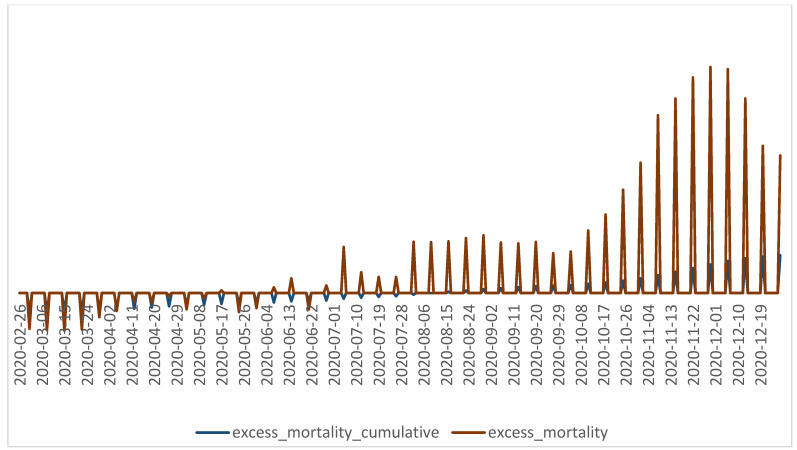
The evolution of the cumulative excess mortality and excess mortality indicators in Romania during March 2020–31 December 2020 [28].

Non-pharmacological measures and their application periods were retrieved from the administrative decisions targeting the public health domain issued in the period between March 2020 and December 2020. Information regarding the evolution of the pandemic in Romania was retrieved from the Our World in Data website (Figure 2 and Figure 3) [28].

The technical committee that managed the NPMs to combat the pandemic in Romania systematized the measures into three categories:

- Measures to increase the response capacity: limiting economic activities, organizing the activity of operators with more than 50 employees, closing schools, limiting internal and international movement, banning public and private events in closed spaces, and limiting the number of participants in public or private events in open spaces with the observance of individual hygiene rules (wearing a mask, washing hands and physical distancing).
- Measures to increase the resilience of the population: Initial quarantine in spaces made available free of charge by the authorities with the provision of food and some items for personal hygiene, then at a chosen residence. In both situations, paid holidays were granted for the employed persons during the quarantine period.
- Measures to reduce the type of risk: Organizational measures at the level of the public authorities, public health and social assistance services. For employees in the health system, free testing and free accommodation spaces were provided to isolate themselves from family members; for workers in nursing homes and social assistance units with accommodation, bimonthly testing and maintenance in the units for an interval of 14 days were provided with strict restrictions of access to outsiders.

During the studied period, these measures were imposed and lifted depending on the evolution of the pandemic.

We started from the following research hypotheses:

**Hypothesis** **1** **(H1).** *The evolution of the pandemic correlates with the measures to increase the response capacity.*

**Hypothesis** **2** **(H2).** *The evolution of the pandemic correlates with measures to increase population resilience.*

**Hypothesis** **3** **(H3).** *The evolution of the pandemic correlates with measures to reduce the type of risk.*

The measures were analyzed by category, but also individually regarding the correlation with the relevant indicators for the evolution of the pandemic: the number of new cases of COVID-19, the reproduction rate and the number of deaths. The administrative acts imposed or intensified certain measures depending on the evolution of the pandemic, and for a temporal correlation with the evolution of the pandemic, we kept the official classification from the studied period. The assignment of values for the measures taken was carried out according to the intensity of the measures throughout the studied period. In order to increase the restrictions, the value attributed to the respective measure was increased during the period in which the measure was imposed. For the periods when the measure was fully or partially lifted, the value was reduced accordingly to respect the temporal disposition of the measures.

Depending on the evolution of the number of cases—initially from the national level and then from the local level—the threshold at which the measures were applied and their severity varied within the period studied, March 2020–December 2020, such that for the impact to be quantified, the measures were codified as follows:

Scenarios involving school function: onsite classes—0, mixed classes (50% of students onsite, 50% online)—1, online classes—2.

Internal travel: no restrictions—0, restrictions for people to travel within the interval of 22:00–5:00—1, restrictions in specific categories at specific time intervals for accessing essential product vendors—2, restrictions pertaining to the whole population—3.

International travel: no restrictions—0, restrictions on entering the country for people who have traveled to countries classified within the yellow or red list—1, suspension of flights from within the country due to an increased COVID-19 incidence—2.

Public and private events in open spaces with the observation of several personal hygiene rules (wearing masks, hand washing and social distancing): no restrictions—0, limiting the number of participants to 200 persons—1, to 100 persons—2, to 50 persons—3, to 20 persons—4, forbidden—5.

Limiting the number of participants at public and private events in closed spaces with the observation of several personal hygiene rules (wearing masks, hand washing and social distancing): no restrictions—0, limiting the number of participants to 200 persons—1, to 100 persons—2, to 50 persons—3, to 10 persons—4, forbidden—5.

Quarantine of 14 days at a chosen residence—1, quarantine for 14 days at a location given by authorities without COVID-19 testing—2, quarantine for 14 days at a location given for free by authorities with COVID-19 testing on the 2nd day and a repeat test on the 12th day—3.

Face mask: no recommendation—0, imposed in closed spaces—1, imposed in open spaces and crowded areas—2.

Hand hygiene: no recommendation—0, recommended when participating at events—1.

Decisions taken: at the national level—1, at the local level—2.

Correlations between non-pharmacological measures and the reproduction rate with regard to the number of new corrected cases were made using the IMB SPSS Statistics application with a Pearson correlation and Bayesian analysis [29].

## 3. Results

This study describes the way in which the pandemic was managed in Romania, analyzing not only the organization of the health system and the adaptation of the health system towards the medical necessities called for due to the evolution of the pandemic, but especially the efficiency of non-pharmacological measures adopted through administrative acts. The impact of the NPMs were analyzed according to the number of COVID-19 cases, the number of deaths and the rate of infection of the disease.

The following statistical analysis showed that the best correlations between measures and the reproduction rate of the disease are for social distancing, hand hygiene and wearing masks. With all of these, the suggestive Bayes factor was calculated for travel restrictions, job activities, online schooling and population travel restrictions within localities (Table 1).

Moderate correlations were found between the measures to increase the response capacity and the number of deaths as follows: closing schools, wearing a mask, passing decisions at the central level to the local level, banning public events and limiting movement.

Thus, for the reproduction rate, linear regressions of the Pearson coefficient were obtained for the following measures: physical distancing (−0.542), hand hygiene (−0.542), wearing a mask (−0.421), and passing decisions from the central to local level (−0.340). Regarding the number of new cases, linear regressions of the Pearson coefficient were obtained for the following measures: movements (−0.014) and lockdown (−0.007). For the indicator of the number of deaths, regressions were obtained for the following measures: lockdown/alert status (−0.269) and limitation of movement (−0.054), Table 2.

By categories of measures, we can observe linear regressions for the reproduction rate of the disease due to the measures for reducing the type of risk (−0.187 Pearson correlation value), the measures for increasing the response capacity (−0.535 Pearson correlation value), as well as those for increasing population resilience (−0.538 Pearson correlation value). For the measures aimed at reducing the type of risk, the correlations are weak. The moderate correlation coefficients observed for these measures can be explained by the fact that in the management of an infectious disease, the measures aimed at isolating the patient, the gate of exit and entry into the body, and the route of transmission cannot be sufficient without measures to reduce the receptive population through immunization.

None of the non-pharmacological measures had a strong correlation with the monitored indicators; moreover, the measures with a major impact on the economy by limiting activities had a low correlation coefficient. Additionally, physical distance had a low correlation coefficient with the number of new cases and the number of deaths. These low correlation coefficients can be explained by the airborne transmission of the disease, a way of transmission that allows an easy spread without necessarily being dependent on the mobility of the infected person.

The number of ICU beds occupied in the hospitals and the excess mortality greatly depend on who is infected and the viral load (age, co-morbidities, etc.). An in-depth analysis with better profiling of the patients would be needed to extract any conclusions from those figures.

Since the number of occupied ICU beds and the number of deaths are indicators that can be influenced by factors such as pre-existing medical conditions or age, they were evaluated in the context of the number of tests and the number of cases. Using the Bayesian analysis of the Pearson correlation for all four indicators, a linear correlation can be observed with the measures taken (Table 3).

## 4. Discussion

The management of the pandemic through non-pharmacological measures was the international standard until the COVID-19 vaccines were available [30]. Lockdowns were an effective measure in stopping the spread of the virus in the short term [31]. It is worth mentioning that the statistical significance that resulted from our study was only from the calculation of measures decreasing the impact of the disease: travel restrictions, restriction of the movement of the population, restriction of public events and the reduction of non-essential economic activities. Regarding the number of new cases, the most important correlations were found in regard to online schooling, wearing masks and the transference of decision making from the national level to the local level. As for what the Bayes factor shows, the highest values are for lockdowns and travel restrictions. Regarding mortality, the strongest correlations were also recorded in regard to online schooling, wearing masks and the transference of decision making from the national level to the local level. The Bayes factor had important values for travel restrictions, social distancing and personal hygiene measures. These results are not proof of the effectiveness or ineffectiveness of the measures taken, since indicators such as the reduction in disease transmission or the reduction in mortality are influenced by multiple factors, the most important being the population’s compliance with the measures. The large number of fines used as a means of constraining the population or economic agents to comply with the imposed measures shows that, in fact, the compliance of the population was reduced. The annulment by the Romanian Supreme Court of Justice of the applied fines led to the reduction to an even greater extent in the compliance with the measures recommended by the medical authorities. Even under these conditions, for some measures a regression of the monitored pandemic evolution indicators was obtained. This fact can be considered an argument in favor of the use of non-pharmacological measures in combating the pandemic. For the measures for which no regression was obtained, but only correlation, we believe that these results can be an argument in favor of a reaction to adapt administrative measures to the evolution of the pandemic, with the lack of a regression in statistical terms being justified by the multifactorial nature of the evolution of the pandemic, including the large number of the receptive population and, especially, the variants developed by the virus that also led to reinfections.

According to the analysis carried out in 131 countries by Li. Y. [32], the measures taken in Romania were similar to those in other countries: closing schools, limiting the movement of the population and reducing non-essential economic activities. We considered that the particularities of the evolution of the pandemic on the territory of a country also come from the way decisions are made (at the central or local level) and the response capacity of the health system. In this context, Romania is not an exception, and the results can be taken into account in the management of any airborne pandemic, not just the one generated by COVID-19. A study on the effectiveness of non-pharmacological measures in combating the pandemic in China [8,33] showed the importance of measures such as quarantine, reducing mobility and wearing a face mask. Additionally, through the mathematical models presented in the study, the interference with the economic factors and with the population’s compliance with the measures imposed by the authorities was highlighted. In the present study, the limitation of the mobility of the population was correlated with the reproduction rate of the disease or with the number of cases (−0.121 or −0.054, respectively, for the Pearson correlation); these values are close to those obtained in the study by Lin R et al.: −0.0465 for the number of cases.

Another study analyzed non-pharmacological measures in 11 European countries and reported from mathematical models an 81% reduction in the number of cases through non-pharmacological measures [34]. The results of our study do not support these data. The study conducted by Yu L. et al. showed also that the measures taken individually do not have a significant impact on reducing the number of cases.

The results of this study, together with other international studies [8,35], show the importance of non-pharmacological measures in managing a pandemic in which there is no specific treatment or vaccine. The experience gained by managing this period of the pandemic may be of use in future confrontations in the development of pandemics through other micro-organisms [36].

Our study had some limitations. First of all, the analysis was limited to the correlations between the evolution of the pandemic and the values calculated for the non-pharmacological measures established by the administrative acts, as we were unable to assess the population’s compliance with these measures. Secondly, in the study, the number of cases and the rate of reproduction, indicators largely dependent on the testing capacity, were very low in the first months and at the end of the studied period. In this way, the correlations with the measures taken are influenced by the test capacity. Thirdly, by using the official data for the reproduction rates of the infection at the national level, references were introduced regarding the local evolution and the measures taken at the local level. However, being a global analysis, we appreciate that this latter limitation did not significantly influence the results of our study.

## 5. Conclusions

Based on the results obtained, we can conclude that non-pharmacological measures had an impact on the evolution of the pandemic in Romania, as linear regressions were found for all three categories of measures in relation to the reproduction rate. The linear regression in terms of the number of new cases and the number of deaths was found only for the lockdown/imposing a state of alert and limiting movement. The positive correlations between the non-pharmacological measures and the studied indicators show a good administrative response to the evolution of the pandemic, but the lack of regression in terms of the number of cases and for most measures in relation to the number of deaths may suggest a low compliance of the population with the measures taken.

## Figures and Tables

**Table 1:** Statistical analysis of the data series regarding the measures to increase the response capacity through the Pearson correlation and the Bayesian method.

		Reproduction Rate	New Cases Smoothed per Million	New Deaths Smoothed
**Lockdown/alert status**	Pearson correlation	−0.232	−0.007	−0.269
	Bayes factor	0.007	21.811	0.001
	N	292	305	285
**Central decisions–local decisions**	Pearson correlation	−0.340	0.501	0.500
	Bayes factor	0.000	0.000	0.000
	N	292	305	285
**Economic activities**	Pearson correlation	−0.137	0.315	0.240
	Bayes factor	1.363	0.000	0.005
	N	292	305	285
**Wearing facial mask**	Pearson correlation	−0.421	0.629	0.669
	Bayes factor	0.000	0.000	0.000
	N	292	305	285
**Hand hygiene**	Pearson correlation	−0.542	0.218	0.090
	Bayes factor	0.000	0.014	6.787
	N	292	304	285
**Public events ban**	Pearson correlation	−0.263	0.416	0.372
	Bayes factor	0.001	0.000	0.000
	N	292	304	285
**Schools closure**	Pearson correlation	−0.166	0.672	0.678
	Bayes factor	0.375	0.000	0.000
	N	292	305	285
**Movements limited**	Pearson correlation	−0.151	0.372	0.377
	Bayes factor	0.775	0.000	0.000
	N	292	305	285
**Travel limits**	Pearson correlation	−0.121	−0.014	−0.054
	Bayes factor	4.119	18.165	13.308
	N	204	217	197
**Physical distancing**	Pearson correlation	−0.542	0.223	0.090
	Bayes factor	0.000	0.010	6.787
	N	292	305	285

**Table 2:** Statistical analysis of data series regarding non-pharmacological measures to combat the pandemic by the Pearson correlation and Bayesian method.

Measure	Statistical Test	Reproduction Rate	New Cases Smoothed per Million	New Deaths Smoothed
**Decrease risk impact**	Pearson correlation	−0.187	0.239	0.163
	Bayes factor	0.127	0.003	0.476
**Increase response capacity**	Pearson correlation	−0.535	0.408	0.554
	Bayes factor	0.000	0.000	0.000
**Community resilience**	Pearson correlation	−0.538	0.491	0.619
	Bayes factor	0.000	0.000	0.000

**Table 3:** Analysis of the measures taken to manage the pandemic and the main indicators of the evolution of the pandemic.

	Bayesian Analysis of Pearson Linear Correlation	Decrease Risk Impact	Increase Response Capacity	Community Resilience
**New deaths smoothed**	Posterior	0.289	0.402	0.475
	95% credible interval	0.218	0.335	0.413
**New cases smoothed per million**	Posterior	0.309	0.264	0.438
	95% credible interval	0.238	0.193	0.375
**ICU patients per million**	Posterior	0.383	0.440	0.649
	95% credible interval	0.314	0.374	0.600
**Total tests per thousand**	Posterior	−0.139	0.802	0.309
	95% credible interval	−0.234	0.763	0.217

## Data Availability

The data presented in this study are available on request from the corresponding author.
